# Pectoralis muscle index might be a factor associated with frailty in older women with breast cancer

**DOI:** 10.55730/1300-0144.5645

**Published:** 2023-04-04

**Authors:** Muhammet Cemal KIZILARSLANOĞLU, Mehmet Ali ERYILMAZ, Betül Çiğdem YORTANLI, İlknur Rahime ÜNAL, Barış Can ÜNAL, Nahide BARAN, Ayşegül ALTUNKESER, Nergis AKSOY

**Affiliations:** 1 Division of Geriatrics, Department of Internal Medicine, Konya City Hospital, University of Health Sciences, Konya, Turkey; 2 Department of General Surgery, Konya City Hospital, University of Health Sciences, Konya, Turkey; 3 Department of Internal Medicine, Konya City Hospital, University of Health Sciences, Konya, Turkey; 4 Department of Radiology, Cihanbeyli State Hospital, Konya, Turkey; 5 Department of Radiology, Konya City Hospital, University of Health Sciences, Konya, Turkey

**Keywords:** Breast cancer, frailty, pectoralis muscle, sarcopenia

## Abstract

**Background/aim:**

To investigate the possible relationship between pectoralis muscle (PM) measurement and frailty in older women with breast cancer (BC) (preoperatively defined as stage 1, 2, and 3 diseases).

**Materials and methods:**

This retrospective, observational study was conducted at Konya Training and Research Hospital between June and December 2020. A total of 102 patients [median age 62.5 years, median follow-up period two years] were included in the study. PM measurements were obtained from thorax computerized tomography (CT). Pectoralis muscle index (PMI) was calculated by dividing the PM area by the height square of the patients (cm^2^/m^2^). Pectoralis muscle density (PMD) was evaluated using CT findings, including their Hounsfield Units (HU). Frailty status and sarcopenia-risk assessments were done by a telephone interview in September 2020 using the FRAIL index (categorized as robust or nonrobust) and SARC-F questionnaire (classified as no sarcopenia-risk or risk of sarcopenia), respectively. PM measurements were compared between robust and nonrobust patients and between patients with a risk of sarcopenia and no sarcopenia risk.

**Results:**

The nonrobust patients had lower pectoralis major muscle index (PMaMI) (p = 0.041) and pectoralis major muscle density (PMaD) (p = 0.020) levels than robust patients in the whole study sample. PMI (p = 0.017) and PMaMI (p = 0.010) levels were significantly lower in the nonrobust patients than in robust patients with early-stage BC. Frailty status was positively correlated with age (rho: 0.621; p < 0.001), BC stage (rho: 0.220; p = 0.026), and SARC-F score (rho: 0.747; p < 0.001), and negatively correlated with PMaMI (rho: −0.197; p = 0.047) and PMaD (rho: −0.237; p = 0.016). There were significant associations between PMaMI (OR: 0.467, 95% Confidence Interval (CI): 0.226–0.962 p = 0.039) and PMI (OR: 0.543, 95% CI: 0.299–0.986 p = 0.045) levels with frailty status (being nonrobust) in regression models.

**Conclusion:**

In the study, it has been shown that pectoralis muscle assessment might be a related parameter to frailty in older women with breast cancer.

## 1. Introduction

In the aging world, life expectancy is also getting older. Moreover, most older people fight against medical problems such as chronic metabolic illnesses, malignancies, and other geriatric syndromes, including dementia, depression, malnutrition, sarcopenia, or frailty [1]. Therefore, these numerous problems precipitate new, unexpected poor clinical outcomes in the older population [2].

Although breast cancer (BC) has higher progression-free and overall survival rates by the effect of new treatment strategies, including targeted therapy modalities, older patients with malignancy are more likely to have a poor prognosis [3]. Determining poor prognostic criteria in a patient with such malignancies will lead to organizing early intervention strategies. Nowadays, frailty and sarcopenia are shown to be two of the significant poor prognostic factors for older people with or without malignancies [4–6]. Both may make an older person more vulnerable to a stressor, even a minor one, with a dramatic decline in their physical and psychological reserves [7,8]. Identifying these geriatric syndromes in a patient before their development will allow for early planning of preventive treatment strategies.

Moreover, contemporary evidence has shown that struggling with frailty and sarcopenia may improve long-term clinical outcomes of older patients. These two entities affect each other; therefore, it can be said that there is a vicious cycle between frailty and sarcopenia [9]. Some clinical investigations show that sarcopenia and frailty are important prognostic factors for malignancies such as gastric, colon, and pancreatic cancers [4, 10–12]. The studies investigating the relationship between frailty and BC have shown that the frailty rate is about 43% and the all-cause mortality rate might be high in frail patients with BC [13, 14].

The cornerstones for diagnosing sarcopenia and frailty are assessing muscle mass, strength, and performance. However, these evaluations are time-consuming and impractical for all clinicians except geriatricians because of needing special devices. Therefore, some practical measurements have been developed to diagnose frailty or sarcopenia [15,16]. One valuable method is measuring a single skeletal muscle mass, including the psoas, pectoralis, or gastrocnemius muscles associated with sarcopenia, with a high correlation rate with total body muscle mass [17]. Additionally, some clinical studies in the literature show that pectoralis, psoas, gastrocnemius, and other muscle assessments significantly correlate with poor prognosis of some diseases, such as gastrointestinal malignancies, chronic obstructive pulmonary disease, or lung cancer [18–20]. However, the number of studies examining these relationships in BC is limited. For example, to our knowledge, no investigation is exploring the association between pectoralis muscle mass measurements and frailty in older BC patients.

Hence, in this study, we aimed to investigate the relationship between pectoralis muscle measurement at the time of BC diagnosis and future frailty status (assessed after a long-time from the diagnosis of BC).

## 2. Methods

### 2.1. Study design and participants

This study had a retrospective, observational design. It was conducted at Konya Health Application and Research Center, a tertiary hospital that is one of our city’s big health centers, between June and December 2020. Before starting the study, all procedures that required approval were obtained.

**Figure 1:**
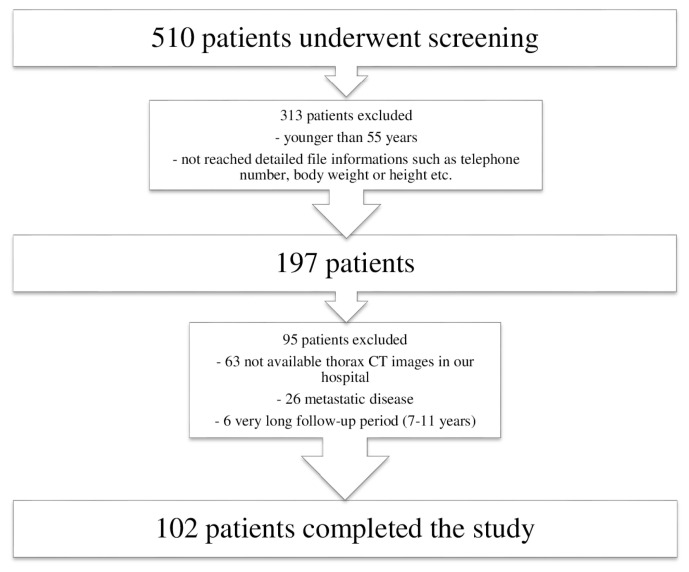
This flow chart shows how the patients were selected.

The patients were selected in our database created between 2008 and 2020, consisting of BC patients evaluated by our hospital surgery committee. Frailty and risk of sarcopenia were assessed by an internal medicine physician (B.C.U) via a telephone interview in September 2020. The patient selection procedure is presented in Figure 1. After investigating 510 patients for eligibility criteria, 102 older women were included in the study. The patients with metastatic disease at BC diagnosis or within the follow-up period, younger than 55 years old, and no thorax computed tomography (CT) imaging to measure pectoralis muscle area (PMA) before BC operation were excluded from the study.

On the other hand, the patients who had Alzheimer’s disease and other dementia diagnoses or could not give information when interviewed by telephone about frailty and sarcopenia risk assessments were also excluded. The patients who had thorax CT scan results and long-term follow-ups were included in the study. FRAIL index and SARC-F scale were applied to the patients 1–4 years after BC diagnosis. These evaluations were done once for all included patients and the reciprocal relationship between PM measurements and FRAIL index and SARC-F score was investigated. Six patients were excluded due to having a very long follow-up interval (7–11 years), when compared to the other patients, between BC diagnosis and frailty and risk of sarcopenia evaluations.

First, the patients were evaluated with a form including questions about demographic characteristics, diagnosis time, stage, pathological features of the BC, and comorbidities. Then, the body mass index (BMI) levels of patients were measured by dividing the weight (kg) by the height square of patients (m^2^). The knowledge about BMI levels was learned from both patients and patients’ files that existed in our hospital. Height and weight, preoperatively measured and noted in the patient’s files in the hospital, were used to calculate the preoperative BMI levels of the patients. In addition, the current BMI levels of the patients were learned from the patients’ files or statements when interviewing.

Then, pectoralis muscle assessments were done by radiologists (N.B. and A.A.) experienced in this field and blind to patients’ frailty and sarcopenia status via CT imaging attained at the BC diagnosis before operation; afterward, the patients were evaluated for frailty status and sarcopenia. Therefore, patients’ frailty or sarcopenia assessments were done at a time interval after BC diagnosis. To clarify the overall, all-cause, and long-term association between PMA assessment and frailty or sarcopenia-risk status, the patients having at least a one-year time interval from the diagnosis of BC to the frailty or sarcopenia evaluation time were included in this study.

### 2.2. Pectoralis muscle area assessment/CT scan analysis

**Figure 2:**
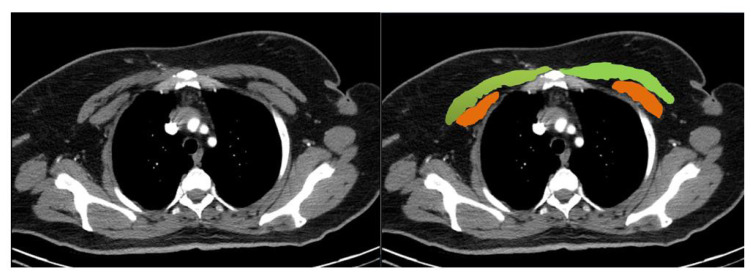
Pectoralis muscle areas were manually measured by the level above the aortic arch. Pectoralis major and minor muscles are shown by green and orange colors, respectively, in this image.

CT scan analyses were performed using Siemens Biography 16 Positron emission tomography/CT (PET/CT) and Philips Brilliance V2 6.1 (2007) devices. All measurements had a 2.5 or 5 mm slice thickness with automatic 120 kVp, 1.25 pitch factor, and mAs scanning parameters. A single axial slice above the aortic arch was quantitatively taken from CT evaluation (Figure 2). Two radiologists experienced in the breast radiology field with more than four years measured patients’ PM areas using the software installed in our radiology department. Bilateral pectoralis major and minor muscles’ areas and densities were quantified using the region of interest. Both pectoralis major and minor muscle areas were summed to determine the pectoralis muscle area. Five different parameters were calculated for each patient:

1. Pectoralis muscle index (PMI): Firstly, the right and left pectoralis major and minor muscles were measured. All measured areas were summed as cm^2^; then, the calculated level of total pectoralis muscle area (PMA) was divided by 2 to obtain the mean PMA level of each patient, and lastly, the received PMA level was divided by the height square of patients to calculate PMI (cm^2^/m^2^).
2. Pectoralis major muscle index (PMaMI): Similar to the calculation of PMI, PMaMI was obtained by dividing the mean pectoralis major muscle area by the height square.
3. Pectoralis minor muscle index (PMiMI): PMiMI levels of the patients were also calculated by dividing the mean pectoralis minor muscle area by the height square described above.
4. Pectoralis major density (PMaD): Pectoralis major muscle density was evaluated using CT findings, including their Hounsfield Units (HU). First, right and left PMaDs were measured and summed; then, total density was divided into two to reach the mean level of PMaD. Finally, the estimated mean PMaD level was considered the patients’ PMaD value.
5. Pectoralis minor density (PMiD): As measured PMaD level, PMiD was assessed similarly.

### 2.3. Evaluation of the frailty status of patients

An internal medicine physician assessed frailty via a telephone interview in September 2020. Since the devastating pandemic worldwide, we could not invite patients to the hospital to learn about their frailty or sarcopenia status. The median follow-up time between the CT date, measured PMI and PMD levels, and frailty assessment date were two years (minimum-maximum: 1–4 years). The frailty assessment was performed using the FRAIL scale, translated into Turkish by Benazir et al. [21]. This scale consists of five self-reported parameters (Fatigue, Resistance, Ambulation, Illnesses, and Loss of weight). If a person has zero point from this evaluation, this one is considered robust, while taking 1–2 points are considered prefrail, and three or more points is frail. Our patients are categorized as robust if they take 0 points and nonrobust if they have one or more points from the FRAIL index.

### 2.4. Evaluation of sarcopenia-risk

The SARC-F scale assessed sarcopenia risk by the same physician with a telephone interview. This scale detects patients who are at increased risk of sarcopenia. After determining the patients with sarcopenia risk, they should undergo further evaluation for a definitive diagnosis. These additional appraisals consist of muscle strength, mass, and performance measurements. Unfortunately, the pandemic did not allow us to evaluate sarcopenia with more invasive methods by inviting patients to the hospital.

Nevertheless, we assessed their sarcopenia risk using the SARC-F questionnaire [22]. This scoring system investigates the patient’s ability to assist in walking, rising from a chair, climbing stairs, strength, and falls by asking self-reported questions. Each item is scored as 0–2 points with a total score ranging from 0 to 10 points. A person with four or more points from this questionnaire was considered at risk of sarcopenia [23].

### 2.5. Ethical statement

Ethical approval was taken from KTO Karatay University Ethical Committee with a decision number and date of 41901325-050.99 and 22 May 2020. In addition, verbal informed consent was taken from the patients to include in the study when interviewing by phone.

### 2.6. Statistical analysis

Statistical Package for the Social Sciences (SPSS) program, IBM, 21.0 version was used for the statistical analyses. Continuous variables were first investigated to determine whether they had skew or normal distribution by evaluating the Kolmogorov-Smirnov test, histogram, and variation coefficient. Since no normally distributed numerical parameter was in our data, all continuous variables were presented as median (min-max). Categorical variables were presented as numbers and percentages (n, %). The patients were divided into two groups, robust patients (group 1) and prefrail-frail (nonrobust) ones (group 2), instead of three, the reason that the frail patient number was meager (only three patients). The Mann-Whitney-U test compared numerical parameters between groups 1 and 2. Categorical variables were analyzed between two groups with Chi-square or Fisher-Exact tests where appropriate. Correlation analysis between frailty status and other numerical parameters was done using Spearman’s correlation test. Rho coefficient lower than 0.30 was considered weak, 0.31–0.70 was moderate, and higher than 0.70 was a strong correlation. Binary logistic regression analyses detected the relationship between PMI, PMaMI, and frailty status with crude and adjusted models. Frailty status as an independent variable in regression analyses was categorized into two groups; group 1: robust and group 2: pre-frail or frail (nonrobust). Group 1 (robust) was the reference group in regression models. First, unadjusted or crude binary logistic regression analyses were applied between PMI, PMaMI, and frailty status in model 1. After that, adjusted methods, including the related parameters, were added to the binary regression models. In model-2, all the parameters that significantly differed or had a p-value lower than 0.20 when compared between robust and nonrobust patients were included. In model 3, all parameters included in model 2, except chronic diseases, were added to the binary logistic regression analysis. The further regression analysis information was also presented in the related table as a footnote. Power analyses were done using the OpenEpi version 3.01 program (Andrew G. Dean and Kevin M. Sullivan, Atlanta, GA, USA), with values higher than 0.80. Receiver operating characteristic (ROC) curve analyses were done using MedCalc Statistical Software version 19.0.6 (MedCalc Software, Ostend, Belgium). In the ROC analyses, cut-off points were tested for pectoralis muscle measurements to show their associations with frailty and risk of sarcopenia. Statistical significance was accepted as a p-value lower than 0.05.

## 3. Results

The median age was 62.5 years (55–90), and the most common comorbidities were diabetes mellitus (33.3%) and hypertension (42.2%). The majority of the patients were robust (74.5%) according to the frailty assessment, and only 3 (2.9%) patients were frail, and 23 patients (22.5%) were prefrail. Using the SARC-F score, five patients (4.9%) were at increased risk of sarcopenia. When the patients were divided into two groups risk of sarcopenia (n = 5) and no risk of sarcopenia (n = 97), and the categorical (gender, comorbidities, BC stages, treatment strategies, type of surgery, BC pathology) and continuous (age, BMI, follow-up time, PMI, PMaMI, PMaD, and PMiD) parameters of the study were compared between groups, no significant differences were observed except PMaD and age. The patients with a risk of sarcopenia (27 HU; min-max: 8–27.5) had a lower level of PMaD compared to the patients with no sarcopenia-risk (30.5 HU; min-max: 1.5–50) (p = 0.038). The patients with a risk of sarcopenia (78 years; min-max: 62–90) were older than the patients with no sarcopenia risk (62 years; min-max: 55–79) (p = 0.004). Median age, coronary artery disease, invasive lobular carcinoma (ILC) pathology frequencies, and rate of having a SARC-F score higher than 4 points were observed to be significantly higher in the nonrobust group than others (all had p-value <0.05). General characteristics and other parameters, compared according to frailty status, are presented in Table 1.

Pectoralis muscle assessment results were compared between the nonrobust and robust groups. The median levels of PMaMI and PMaD were significantly lower in the nonrobust patients when compared with robust ones (all had p-value <0.05); additionally, PMI had lower median levels with a borderline significance in the nonrobust patients compared with others in the whole study population (p = 0.087). In addition, significantly lower PMI and PMaMI levels were detected in the nonrobust patients than robust in those with early-stage breast cancer (all had p-value <0.05). In the Table 2, all comparisons of pectoralis muscle measurements according to the frailty status of patients in addition to the BC stages are shown.

Correlation analyses between frailty status (as an ordinal variable) and other continuous variables revealed that frailty was positively and significantly correlated with age (moderate correlation, rho: 0.621), SARC-F score (strong correlation, rho: 0.747), and BC stage (weak correlation, rho: 0.220) while negatively and significantly correlated with PMaMI (weak correlation, rho: −0.197) and PMaD (weak correlation, rho: −0.237) levels. Correlation analysis results are presented in Table 3.

Regression analyses, including crude and adjusted models, were prepared to show the exact relationships between frailty status and pectoralis muscle measurements. These analyses demonstrated that per cm^2^/m^2^ increase in PMI was associated with about 17% decreased relation to being nonrobust in this study’s follow-up period in crude regression (with a borderline significance); moreover, this rate was found to be approximately 46% when adjusted for major confounding parameters with of importance. Similarly, per cm^2^/m^2^ increase in PMaMI was associated with being nonrobust in crude (p = 0.039) and adjusted regression (p = 0.039) models with decreased relation to 24% and 53%, respectively. Regression analysis results are shown in Table 4.

In the ROC curve analyses, PMI (area under the curve (AUC): 0.613 p = 0.085), PMaMI (AUC: 0.685 p = 0.032, criterion ≤ 4.155, 65% sensitivity and 58% specificity), PMiMI (AUC: 0.503 p = 0.974), PMaD (AUC: 0.653 p = 0.012, criterion ≤ 27.5, 62% sensitivity and 72% specificity), and PMiD (AUC: 0.516 p = 0.816) showed low-level AUCs when indicating the interactions of them with frailty status. When all these analyses were done to show the interactions between the parameters (PMI, PMaMI, PMiMI, PMaD, and PMiD) and risk of sarcopenia in the ROC curves, it was seen all had p-values higher than 0.05 except PMaD (AUC: 0.775 p < 0.001, criterion ≤ 27.5, 100% sensitivity and 67% specificity).

## 4. Discussion

In this study, it has been shown that older women might be at increased risk of being nonrobust (prefrail/frail) in a two-year follow-up time if they have lower pectoralis muscle levels at the time of diagnosis of breast cancer. In other words, the patients being nonrobust after two years from BC diagnosis had lower or worse pectoralis muscle assessment results at the time of BC diagnosis.

Older age is one of the significant risk factors for frailty [24]. Nearly half of the patients aged 90 and older are considered frail [24, 25]. On the other hand, age is not a single predictor for frailty or sarcopenia; therefore, a comprehensive assessment is needed to find exact frail or sarcopenic patients with appropriate assessment methods. Not surprisingly, in our study, the median age was found to be older in the nonrobust group than in robust ones and patients with a risk of sarcopenia compared to no sarcopenia risk. Additionally, the nonrobust patients had a higher coronary artery disease rate than the robust patients in this study. Current evidence has shown that frail patients are more likely to have multimorbidities, including atherosclerotic vascular disease, which has an inflammatory process in its pathogenesis as frailty [26].

Another finding in our study is that the nonrobust group had a higher rate of having invasive lobular carcinoma (ILC) pathology than others. To our knowledge, there is no evidence investigating this relationship before. Further investigation into this subject will lead to an understanding of the interaction between frailty and pathological subtypes of breast cancer. According to this finding, it can be said and speculated that ILC might cause a more aggressive disease course in older adults by leading to frailty compared to patients with invasive ductal carcinoma (IDC) pathology. Since our work was not a prospective study, the causality between the abovementioned parameters could not be said precisely; therefore, prospectively designed studies are needed.

Another significant finding from our study is that sarcopenia risk was higher in the nonrobust group than in the others. It is well-known that sarcopenia and frailty are two common geriatric syndromes, especially in vulnerable older adults; therefore, it will not be false if we say frail patients are at an increased rate of sarcopenia risk or vice versa. Supporting this information, in the recent study, the risk of patients with sarcopenia risk was detected to be higher in the nonrobust group than others, in addition to the findings that showed a significant correlation between frailty status and the SARC-F score. However, as only five patients (4.9%) was at risk of sarcopenia according to the SARC-F scale in this study population, further analyses investigating sarcopenia risk and pectoralis muscle assessment result could not be reached. Moreover, the pandemic era did not allow us to assess both frailty and sarcopenia with a more valuable measurement by inviting patients to the hospital. Nevertheless, although these evaluations were done by telephone interview, we could achieve many significant findings that will light many further studies.

Skeletal muscle assessment with CT or other techniques is a recently preferred method for evaluating sarcopenia and determining the prognosis of certain diseases such as malignancies, chronic obstructive pulmonary disease (COPD), etc. [27, 28]. Psoas and lower limb muscles are widely used for these reasons. Although the studies investigating pectoralis muscle measurements are more limited than those exploring other muscles, some clinical studies are available showing its impact on disease prognosis. For instance, in a study by Kinsey et al., 252 lung cancer patients were evaluated. They found that the CT-derived pectoralis muscle area might be a good indicator for worsening overall survival [19]. Another study including BC patients showed that the pectoralis muscle area calculated preneoadjuvant chemotherapy regimen was larger than the area measured postchemotherapy after a median 4-year follow-up period [29].

Additionally, Diaz et al. investigated and found that low muscle mass (pectoralis or para-vertebral erector spine muscles) was associated with increased mortality in current smokers during a median of 6.5 years of follow-up [30]. Furthermore, another study has demonstrated that PMI might be a predictor for showing low bone mineral density associated with osteosarcopenia [31]. Finally, McDonald et al. showed that PMI was associated with worse morbidity rates in COPD patients [32].

Skeletal muscle mass (SMM) measurement by bioelectrical impedance analysis (BIA) is a hallmark indicator for sarcopenia in clinical assessment. In a study examining the correlation between PMI and SMM, measured by BIA, after exploring 434 subjects, it was found that PMA assessment on a single axial CT imaging showed a significantly positive correlation with total body SMM [33]. Moreover, in a current study conducted by Ufuk et al., they hypothesized to show the prognostic effect of PMI on coronavirus disease-19 and found in their research that PMI level was associated with prolonged hospital stay and increased intubation and mortality rates [34]. Although our study did not reach all muscle measurements, PMI, PMaMI, and PMaD were the parameters that had a significant association with prefrailty/frailty; especially, pectoralis major had shown an impressive association between frailty in older women with breast cancer.

Some of the PM measurements differed between robust and nonrobust ones in our study; however, due to the small sample size, not all parameters had reached significant differences in terms of the stage of BC. Nevertheless, considerable differences have shown there might be an association between PM and frailty in this population. Further prospective studies might offer a more objective causality relation between mentioned parameters. We have found significant differences in PM measurement in the early-stage BC patients; however, there were no differences in locally-advanced BC patients. We think that since the numbers of patients between robust (n = 17) and nonrobust groups (n = 10) in the patients with locally-advanced stage BC were small, the statistical power was lower (might be a beta error); therefore, we could not find significant differences in terms of PM measurements between mentioned groups. Further studies with prospective design will show the most robust findings.

This study has some limitations. First, the number of patients included in the analyses was limited. However, as the study had a single center, this number of cases may be enlarged by studies designed with multicenters. Another limitation is that a telephone interview did frailty and sarcopenia evaluations with the Frail index and SARC-F score; it would be better if a study measures these syndromes with more accurate parameters, including muscle mass, strength, and performance assessments with suitable devices. However, the pandemic could not allow us to evaluate these parameters mentioned by inviting the patients to the hospital. Another point is that chest CT is not routinely recommended for early-stage BC patients; we included the patients who had this imaging results; we believe that if we have this imaging in these patients or other stages of the disease course, we will have a chance to evaluate PMI levels to predict further prognosis of these patients. The other limitation was that most of the study population was in their sixties, the frailty rate was meager, and only three patients were frail according to the FRAIL index. Therefore, the studies including more frail patients with BC will show valuable findings about the study questions. Lastly, although the patients were followed up for 1–4 years, further studies having a much more follow-up period would show more accurate results between PMI and frailty or sarcopenia in older women with breast cancer. Since we did not know the patients’ frailty status and sarcopenia risk levels at the time of BC diagnosis, we could not find a causal effect between the study parameters. Our findings have emphasized that there was a reciprocal relationship between PM measurement and all-cause frailty in the study period. This study could not show causality between PM measurement and frailty due to the lack of longitudinal assessment of frailty status and not prospective design. Further studies with prospective design should be done to clarify this point. Nevertheless, to the best of our knowledge, since our study was the first on this subject, we believe our findings will shed light on future studies.

In conclusion, older women who were nonrobust after about two years from BC diagnosis might have lower pectoralis muscle index measured at the time of the disease diagnosis than robust ones. Therefore, it has been shown that pectoralis muscle assessment might be related to the frailty of older women with BC in the study.

## Figures and Tables

**Table 1:** General characteristics and comorbidities of the patients according to the frailty status.

Parameters	Total patients n = 102	Pre-frail or frail patients n = 26	Robust patients n = 76	p-value
Age, years	62.5 (55–90)	72.5 (58–90)	60.5 (55–73)	**<0.001**
Gender n (%)				
Female	102 (100.0)	26 (100.0)	76 (100.0)	1.000
BMI at the time of diagnosis of breast cancer, kg/m^2^	31.64 (25.56–44.22)	31.83 (26.56–42.97)	31.64 (25.56–44.22)	0.634
BMI at the end of follow-up time, kg/m^2^	32.05 (26.37–42.60)	32.04 (27.70–41.02)	32.05 (26.37–42.60)	0.642
Comorbidities n (%)				
• Diabetes mellitus	34 (33.3)	10 (38.5)	24 (31.6)	0.520
• Hypertension	43 (42.2)	14 (53.8)	29 (38.2)	0.162
• Coronary artery disease	6 (5.9)	5 (19.2)	1 (1.3)	**0.004**
• Hyperlipidemia	4 (3.9)	3 (11.5)	1 (1.3)	0.050
• Atrial fibrillation	2 (2.0)	1 (3.8)	1 (1.3)	0.447
• Hypothyroidism	5 (4.9)	1 (3.8)	4 (5.3)	1.000
Breast cancer stage n (%)				
• Stage 1	37 (36.3)	5 (19.2)	32 (42.1)	0.085
• Stage 2	38 (37.3)	11 (42.3)	27 (35.5)	
• Stage 3	27 (26.5)	10 (38.5)	17 (22.4)	
Chemotherapy				
• Neo-adjuvant	41 (40.2)	14 (53.8)	27 (35.5)	0.100
• Adjuvant	61 (59.8)	12 (46.2)	49 (64.5)	
Radiotherapy	102 (100.0)	26 (100.0)	76 (100.0)	-
Hormone-therapy	81 (79.4)	23 (88.5)	58 (76.3)	0.186
Type of surgery				
• Modified radical mastectomy and/or lymph node dissection	77 (75.5)	14 (53.9)	63 (82.9)	**0.008**
• Simple mastectomy	23 (22.5)	11 (42.3)	12 (15.8)	
• Quadranectomy	2 (2.0)	1 (1.3)	1 (3.8)	
Breast cancer pathology n (%)				
• IDC	90 (88.2)	19 (73.1)	71 (93.4)	**0.011**
• ILC	7 (6.9)	7* (26.9)	5* (6.6)	
• Other	5 (4.9)			
Sarcopenia risk according to SARC-F score	5 (4.9)	5 (19.2)	0 (0.0)	**0.001**
Follow-up time, years	2 (1–4)	2 (1–4)	2 (1–4)	0.880

BMI: Body mass index; IDC: Invasive ductal carcinoma; ILC: Invasive lobular carcinoma; SARC-F score ≥4 points is considered as sarcopenia risk; Continuous parameters were presented as median (min-max);

* the patients with ILC and other pathology were considered within the same group when comparing between groups.

**Table 2:** Pectoralis muscle assessment results were compared between the robust and nonrobust patients.

Parameters	Total patients	Prefrail or frail patients	Robust patients	p-value
**Whole patients**				
	**n = 102**	**n = 26**	**n = 76**	
PMI, cm^2^/m^2^	5.68 (1.51–13.46)	4.99 (1.51–9.20)	5.86 (2.45–13.46)	0.087
PMaMI, cm^2^/m^2^	4.44 (0.77–12.06)	3.78 (0.77–7.85)	4.73 (1.94–12.06)	**0.041**
PMiMI, cm^2^/m^2^	1.20 (0.43–2.29)	1.10 (0.43–2.18)	1.23 (0.48–2.29)	0.969
PMaD, HU	30.5 (−1.5–50.0)	27.0 (8.0–42.0)	31.75 (−1.5–50.0)	**0.020**
PMiD, HU	28.0 (4.5–56.5)	29.5 (4.5–56.5)	27.3 (6.0–52.0)	0.809
**Patients with early-stage breast cancer**				
	**n = 75**	**n = 16**	**n = 59**	
PMI, cm^2^/m^2^	5.69 (2.52–12.14)	4.77 (2.65–9.07)	5.99 (2.52–12.14)	**0.017**
PMaMI, cm^2^/m^2^	4.56 (2.04–10.50)	3.55 (2.07–7.13)	4.93 (2.04–10.50)	**0.010**
PMiMI, cm^2^/m^2^	1.19 (0.43–2.17)	1.04 (0.43–2.17)	1.26 (0.48–1.99)	0.244
PMaD, HU	31.0 (−1.5–50.0)	26.5 (8.0–42.0)	32.5 (−1.5–50.0)	0.057
PMiD, HU	28.0 (4.5–46.5)	30.5 (4.5–37.5)	27.0 (6.0–46.5)	0.712
**Patients with locally-advanced stage breast cancer**				
	**n = 27**	**n = 10**	**n = 17**	
PMI, cm^2^/m^2^	5.45 (1.51–13.46)	5.89 (1.51–9.20)	5.15 (2.45–13.46)	0.616
PMaMI, cm^2^/m^2^	3.92 (0.77–12.06)	4.46 (0.77–7.85)	3.77 (1.94–12.06)	0.920
PMiMI, cm^2^/m^2^	1.23 (0.49–2.29)	1.52 (0.65–2.18)	1.22 (0.49–2.29)	0.228
PMaD, HU	30.0 (2.0–39.0)	27.5 (18.0–38.0)	30.50 (2.0–39.0)	0.217
PMiD, HU	31.5 (7.0–56.5)	28.0 (13.5–56.5)	33.0 (7.0–52.0)	0.900

PMI: Pectoralis muscle index; PMaMI: Pectoralis major muscle index; PMiMI: Pectoralis minor muscle index; PMaD: Pectoralis major density (muscle radiation attenuation); PMiD: Pectoralis minor density (muscle radiation attenuation). The numerical parameters were presented as median (min-max). HU: Hounsfield Unit.

Early-stage breast cancer was defined as patients with stage I, IIA, or a subset of stage IIB (T2N1) disease, while locally advanced breast cancer including patients with stage IIIA, IIIB, IIIC, and a subset of stage IIB (T3N0).

**Table 3:** Correlation levels of the continuous variables with frailty statuses.

Parameters	rho-coefficient	p-value
PMI, cm^2^/m^2^	−0.164	0.099
PMaMI, cm^2^/m^2^	−0.197	**0.047**
PMiMI, cm^2^/m^2^	0.013	0.901
PMaD, HU	−0.237	**0.016**
PMiD, HU	0.025	0.799
Age, years	0.621	**<0.001**
Breast cancer duration, years	−0.008	0.939
Breast cancer stage	0.220	**0.026**
SARC-F score	0.747	**<0.001**

PMI: Pectoralis muscle index; PMaMI: Pectoralis major muscle index; PMiMI: Pectoralis minor muscle index; PMaD: Pectoralis major density; PMiD: Pectoralis minor density. Frailty status was considered robust (0), prefrail (1), and frail (2) as ordinal variables. Breast cancer stage was defined as stage 1 (1), stage 2 (2), and stage (3) as an ordinal variable.

**Table 4:** Relationships between pectoralis muscle measurement results and frailty status in regression models

Parameters	OR	95% confidence interval	p-value
PMI^1^§	0.822	0.661–1.023	0.079
PMI^2^§	0.543	0.299–0.986	**0.045**
PMI^3^§	0.722	0.510–1.002	0.066
PMaMI^1^§	0.758	0.584–0.986	**0.039**
PMaMI^2^§	0.467	0.226–0.962	**0.039**
PMaMI^3^§	0.657	0.434–0.994	**0.047**

PMI: Pectoralis muscle index; PMaMI: Pectoralis major muscle index; OR: Odds ratio

1 Model 1: Unadjusted or crude binary logistic regression.

2 Model 2: Adjusted for age, hypertension, coronary artery disease, hyperlipidemia, breast cancer stage, surgery type, adjuvant or neoadjuvant chemotherapy usage, and pathological type. This model included the parameters significantly different or with a p-value lower than 0.20 in univariate analysis between robust and nonrobust patient groups.

3 Model 3: Adjusted for age, breast cancer stage, surgery type, adjuvant or neoadjuvant chemotherapy usage, and pathological type. Chronic diseases were excluded from this model because the FRAIL scale, which evaluates chronic disease numbers, may be affected by these disorders when assessed by binary logistic regression analysis.

Frailty status as an independent variable in regression analyses was categorized into two groups; group 1: robust and group 2: prefrail or frail (nonrobust). Group 1 (robust) was the reference group in binary logistic regression models.

§ per cm^2^/m^2^ increase in pectoralis muscle measurements.
